# The IgE repertoire in patients with food allergy resolved at component level

**DOI:** 10.1186/2045-7022-3-S3-P96

**Published:** 2013-07-25

**Authors:** S Belohlavkova, M Fuchs, L Mackova, I Svarcova

**Affiliations:** 1 Immuno-flow, Prague, Czech Republic

## Background

Currently, more than 1200 purified allergens with defined structures are available. Some of them are used for food allergy diagnostics. We aimed to assess individual patients´sensitizationtization profiles of food allergens by means of component resolved diagnosis.

## Methods

Serum samples were analysed by microarray Immuno CAP ISAC (Phadia), which allows to detect specific IgE against more than 100 of molecular components. We have retrospectively analyzed 153 patients with proven food allergy, 96 of them being children under 18 years. We recorded occurence of early reactions including OAS and anaphylaxis and also delayed reactions (atopic eczema and eosinophilic gastrointestinal diseases). IgE antibodies against individual food alergens were divided to groups based on their origin and affiliation to any of panalergen families. With this approach, antibodies against ten groups of alergens were analyzed in individual age categories and diagnostic groups: cow’s milk allergens, hen’s egg allergens, parvalbumins, lipid transfer proteins, flour allergens including gluten, vicilins, conglutins, cupin family allergens, 2S albumins and PR-10 family proteins.

## Results

As expected, in patients below age of 3 years, allergens to mild and egg occured most frequently. Suprisingly, however, we detected more than 40% of patients being sensitization against seed storage proteins (2 s albumins) and almost 30 % of patients have been sensitized by alergens of PR-10 group. In children allergic to peanut sensitization to proanafylactis alergens (Arah h 1, 2, 3) prevailed. In contrary, in older patients over 18 years we have found mainly sensitization to Ara h 8 from Bet v 1 family homologous proteins. Patients with eosinophilic gastrointestinal diseases, mainly eosinophilic esophagitis, demonstrated sensitization to Bet v 1 homologous proteins in more than 50% and almost 30% sensitization to allergens from seed storage protein families. Approximately 20% of our patients had detectable antibodies against one or more of lipid transfer proteins.

## Conclusion

CRD using microarray technics enables to detect IgE sensitisation profile not only in one patient, but also in a group of patients with defined age and diagnosis. This fact could contribute to optimalisation of diet according to possible cross-reactivity. Sensitization to proanaphylactic alergens, like LTP´s and seed storage proteins, enables to detect individual risk of food induced anaphylaxis.

## Disclosure of interest

None declared.

